# HIV stigma in Romania – from the generation of nosocomially-infected children to the new generation of injecting drug users. Results from a qualitative study

**DOI:** 10.1186/1471-2334-14-S7-O5

**Published:** 2014-10-15

**Authors:** Florin Lazar

**Affiliations:** 1 Faculty of Sociology and Social Work, University of Bucharest, Romania

## Background

As a result of treatment advances, HIV is a chronic condition in Romania, but stigma is still a challenge for people living with HIV (PLHIV). More than half of all registered PLHIV were nosocomially-infected between 1988-1990, followed by adults sexually-infected, but in the last three years HIV infection exploded among injecting drug users (IDU). The objective of the present study was to explain the variations of HIV stigma in these three different social groups and the coping strategies used to be resilient.

## Methods

Thematic analysis was performed based on twenty in-depth interviews with PLHIV from three groups: (G1) those from the generation ‘88-’90, (G2) PLHIV infected as adults and (G3) the new group of HIV+IDU.

## Results

At different levels, stigma is present among all groups. In G1, stigma was experienced more severely in the early years of the infection more by the family and less sensed by themselves; now they tend to hide their status developing a jargon/encrypted language in the communication with members of the same group (e.g. on social media such as Facebook) to prevent stigma.

In G2 on one hand there are people with a longer history of the infection who experienced stigma after disclosure now being reluctant to further disclose and on the other hand there are those who stay in secrecy (sometimes even from family members), trying to continue their life as if HIV is not present.

For G3 beside HIV stigma (anticipated and internalized) appears one associated with drug use or other addictive behaviors (e.g. alcoholism), sometimes even from other PLHIV (e.g. from G1 and G2) or from healthcare staff.

The resilient ones are those managing better their stigma (i.e. controlled disclosure), who have family support and are socially active in G1 and G2 while those overprotected by the family from G1 strive for normality (wish to have a family, children, a job). In G3 family, peers and religious beliefs act as resilience factors.

## Conclusion

Health and psychosocial professionals need to understand how stigma impacts differently the life of their patients/clients, what are the triggers for resilience and how to adjust their interventions to optimize results.

